# Supplementary material for: Rapid assessment of noma: Reporting on forgotten and neglected disease in Ethiopia
Source: PLoS Negl Trop Dis. 2024 Dec 13;18(12):e0012696. doi: 10.1371/journal.pntd.0012696 (PMC11676800; doi:10.1371/journal.pntd.0012696)
Supplement: Attachment — Submitted filename: Response to review 1292024.docx [file pntd.0012696.s002.docx]

Dear reviewer

Thank you very much for the constructive feedback. To respond point by point for the comment provided:

1. Author summery (178 words) has been added between the abstract and the introduction
2. The data availability statement has been revisited as “All data are in the manuscript and/or supporting information files"
3. For figure 1, we used QGIS, and the source of map data is plotted in https://www.openstreetmap.org and contributors.
4. For figure 2, has been removed since I could not get a response from the author for the use
5. [Figure2] English consent: attached the English translation (from Amharic)

Thank you again
