# Supplementary material for: Rapid assessment of noma: Reporting on forgotten and neglected disease in Ethiopia
Source: PLoS Negl Trop Dis. 2024 Dec 13;18(12):e0012696. doi: 10.1371/journal.pntd.0012696 (PMC11676800; doi:10.1371/journal.pntd.0012696)
Supplement: Attachment — Submitted filename: Noma manuscript 8102024_with track change.docx [file pntd.0012696.s003.docx]

**Rapid assessment of noma: Reporting on forgotten and neglected disease in Ethiopia**

Wendemagegn Enbiale^1, 2^*

Affiliations

1. Bahir Dar University, College of Medicine and Health Sciences, Bahir Dar, Ethiopia
2. Collaborative Research and Training Center for Neglected Tropical Diseases, Arba Minch University, Arba Minch, Ethiopia

* Corresponding author

Wendemagegn Enbiale

wendemagegnenbiale@gmail.com

**Abstract**

Background: noma is a rapidly progressing, invasive, and debilitating orofacial disease that affects the most vulnerable and marginalised populations worldwide. The people most at risk of noma are pre-school children, exposed to other risk factors, such as malnutrition and diseases of poverty.

Ethiopia is one of the countries with higher reported cases of noma since 2010 and those are only from medical missions. Evidence on the Burden of the disease or epidemiological data are important for planning and prioritizing service delivery, as well as for formulating disease prevention strategies. In this endeavour to document the presence of noma in Ethiopia, and assess the health system capacity for noma care, we have performed a rapid assessment.

Methodology: we performed a rapid assessment using a desk and literature review, health sector capacity assessment and a retrospective assessment to identify all confirmed cases of noma cases based on 2015-2022 data from NGOs and health facility records.

Result: From the desk review it was found that there is no standalone national policy on noma or NTDs in Ethiopia. However, the health policy of the country emphasizes the prevention and control of diseases of poverty. And no written oral health program for primary healthcare except the availability of dental care in limited regional/referral hospitals and private sector. The retrospective assessment has extracted 69 noma cases record reported from January 2015 to December 2020 and 97% of the record came from two NGO’s who are supporting the surgical mission. Cases are from almost every corner of the country with strong focality in Amhara region, the trend of cases being cared has decreased from 2015 to 2020. No record is found for acute cases of noma.

Conclusion: this rapid surveillance has identified a striking scarcity of research and surveillance programmes considering noma. Intensify efforts in terms of public awareness and education of community workers and health professionals at existing health centres on identification of Noma and subsequent referral of patients to referral hospitals. The assessment emphasizes that a first step to noma elimination should be the inclusion of noma in the national list of neglected tropical diseases, followed by broad-based integrated control programmes using the existing health extension system in expanding oral health.

**Authors summery**

Noma is a rapidly progressing and debilitating orofacial disease, primarily affecting vulnerable, malnourished children in impoverished regions. Ethiopia has seen increased cases of Noma since 2010, mainly through medical missions. Understanding the disease's burden is essential for planning and prevention.

To assess Noma in Ethiopia, we conducted a rapid evaluation, including literature reviews, health sector assessments, and a retrospective analysis of confirmed cases from 2015-2022. Our findings show that Ethiopia lacks a standalone national policy on Noma, though the national health policy addresses poverty-related diseases. There is also no formal oral health program within primary care; dental services are limited to regional hospitals and the private sector.

The review identified 69 Noma cases reported between 2015 and 2020, mostly from two hospitals in the capital supported by NGOs. Cases were found across nearly all regions, with a concentration in Amhara. No acute Noma cases were recorded, and overall cases declined over the years.

This assessment underscores the need for enhanced public awareness, better training for health workers, and integrated control programs to address Noma through existing health systems.

**Introduction**

Noma, also known as cancrum oris or gangrenous stomatitis, is a debilitating and progressive orofacial disease that affects marginalized populations worldwide. It is a non-communicable and invasive condition often referred to as the "face of poverty." Noma primarily targets children between the ages of 2 and 6, although there have been reported cases in immunocompromised adults as well (1, 2).

The exact cause of noma remains unknown, but it is believed to stem from a combination of factors, including malnutrition, immunocompromisation (such as systemic bacterial or viral infections, immunosuppression associated with HIV, measles, malaria, tuberculosis, leukemia, or non-Hodgkin lymphoma), concomitant diseases like measles or malaria, poor oral hygiene, certain social and environmental factors, damage to the gingival mucosa barrier, and bacterial factors (2-5).

The disease manifests in five stages, with the acute stage encompassing the first three stages, which typically occur within a span of two weeks. If left untreated, noma has an estimated mortality rate of 80-90% (1, 6, 7). Failure to address the disease leads to the progression of necrotising gangrenous infection in the orofacial area (third stage). Survivors experience wound healing and scarring over several months (fourth stage), which can result in facial deformities, trismus, and ankyloses of bones and joints. The final stage, known as noma sequelae, occurs approximately a year later and is characterized by significant functional, visual, and physical disabilities that impede proper nutrition intake and carry a heavy social stigma (7).

Historically, noma was widely reported in Europe and India during the 1800s, as well as in concentration camps during World War II and the war-time population of the Netherlands following the winter famine of 1944/45 (8, 9). However, since the latter half of the 20th century, most noma cases have been documented in low and middle-income countries in Africa, South America, and Asia. Burkina Faso, Ethiopia, Mali, Niger, Nigeria, and Senegal have reported the highest number of cases (9). Unfortunately, many instances of noma go undetected and unreported due to various factors, including limited diagnostic skills among health workers and the general population's inability to recognize the disease, rapid disease progression with high acute-phase mortality, the absence of routine surveillance and health facility information systems that include noma, and families hiding affected children due to the associated social stigma (10).

With only a limited number of individuals able to access treatment during the active phase, our understanding of the disease remains scarce. The most recent global estimate from the World Health Organization (WHO) was conducted in 1999, which projected an annual occurrence of 140,000 new noma cases worldwide, with 770,000 patients living with Noma sequelae at that time. However, the origin and reliability of this estimate are unclear (11). Country-specific estimates have shown an annual incidence of 4.2 acute noma cases per million in Senegalese children (12). A recent retrospective chart review estimated a community-based point prevalence of 33 out of every 1000 children aged 0-15 years in northwest Nigeria (13).

Ethiopia is one of the countries with a higher incidence of the disease, but reports of noma cases have only been documented by medical missions and non-governmental organizations since 2010 (14, 15). Despite the prevalence of reports from medical missions, a desk review revealed a lack of supportive data from the public health information system, making the burden and geographic distribution of noma is not known. In June 2022, the Ethiopian Ministry of Health has expressed interest in joining the WHO Regional noma Control Program (RNCP) for the "sustainable elimination of noma as a public health problem in the African Region". Evidence on the Burden of the disease or epidemiological data are important for planning and prioritizing service delivery, as well as for formulating disease prevention strategies. In this endeavour we performed a rapid assessment with the objectives of understanding the burden and geographic distribution of noma 2) stakeholder engagement in noma prevention and control; 3) Knowledge of health professionals on noma 4) the capacity of the health system to respond to Noma prevention and control.

**Methodology**

**Ethics statement**

The assessment was commissioned by the WHO regional office in collaboration with the Ethiopian Federal Ministry of Health (MOH). We secured a support letter from the MOH for the respective hospitals, granting access to the Health Information Management System and outpatient clinical records. Individual consent was not possible to collect since the study is from retrospective data but to ensure privacy and confidentiality, the data was anonymized for any information that could identify participants.

Given that the project was an integral part of the MOH for health system planning, a formal ethical clearance was deemed unnecessary. Nevertheless, Bahir Dar University's Institutional Review Board granted a waiver for the publication of the data, with Protocol number 785/2023, on September 11, 2023.

**Study design**

We conducted a concurrent triangulation mixed methods (qualitative and quantitative) retrospective cohort study plus literature review and health Sector Capacity Assessment to identify all confirmed cases of Noma cases based on 2015-2022 data from NGOs and hospital record books.

**General Setting**

Ethiopia, the second-most populous country in Africa, has a population of approximately 117,000,000, with 20.9% residing in urban areas. Foreign aid plays a significant role in Ethiopia's GDP, as it is among the top 10 aid recipients globally. Despite being one of the poorest countries, with a per capita income of $960, Ethiopia ranks low in Africa for total health expenditure (16). According to the National Health Accounts (NHA-7) report, Ethiopia's per capita health expenditure in 2016/17 was $33.2 USD, significantly below the recommended $86 USD required to provide basic services in low-income countries (17).

Currently, the country's potential public health coverage is 100%, with a life expectancy of 64 years. The maternal mortality rate (MMR) stands at 412/100,000, the under-5 mortality rate (U5MR) is 55/1,000, and HIV prevalence is 0.9%. The childhood coverage of all basic vaccines is 43.1%, and 37% of children under 5 are stunted, with regional variations ranging from 12% in Addis Ababa to 49% in Tigray. Ethiopia has the lowest health workforce density in sub-Saharan Africa, with only 1.4 health workers per 1,000 population (16,17). The top five risks leading to deaths and disabilities in the country include malnutrition, WaSH (Water, Sanitation, and Hygiene), air pollution, dietary risks, and high fasting blood glucose levels (18).

The Health care system in Ethiopia is decentralized, with three tiers. These include, at the primary care level, is a District-based system that includes a Health post, Health centeres and Primary Hospital. The secondary care level includes General Hospitals to referral hospitals, while the tertiary level provides care at teaching and Specialised Hospitals.

**Data Collection and Analysis**

*Quantitative data collection;* For the retrospective data collection, 13 referral hospitals in six regions and two administrative towns were chosen by purposive sampling. From the selected Hospital one is a private hospital and the other 12 are public hospitals (Figure 1 and Table 1).

Figure 1: Map of Ethiopia, distributions of the selected hospitals selected for rapid assessment of noma in September 2022 (Source: https://www.openstreetmap.org/).

The selection of hospitals was done based on convenience prioritizing institutions that provide plastic surgery, oral care, and dermatology services in each major regions in the country, except Tigray which is not represented in the study since it was not accessible in the data collection period because of the war. Two of the only NGOs (Project Harar and Facing Africa) involved in Noma management and care in the country have been included in the study.

Table 1: Regional and referral hospitals in Ethiopia selected for rapid assessment of noma in September 2022.

|  | **Region** | **Town** | **Hospital** | **Catchment population** |
| --- | --- | --- | --- | --- |
| 1 | Amhara | Bahir Dar | *Tibebe Ghion specialized teaching hospital | 3 to 5 million |
|  |  | Boru Meda | Boru Meda specialized hospital | 1.5 million |
| 2 | Afar | Dubuti | Dubti referral hospital | 1.5 million |
|  |  | Amibara | Mohammed Akile memorial general hospital | 1.5 million |
| 3 | Somali | jigjga | Karama general hospital | 1.5 million |
|  |  |  | Jigjga University teaching hospital | 3 to 5 million |
| 4 | Southern nation nationality people (SNNP) | Arba Minch | Arba Minch general hospital | 1.5 million |
|  |  | Sodo | Wolayita otona referral hospital | 3 to 5 million |
| 5 | Harari | Harar | Hiwot Fana teaching hospital |  |
| 6 | Oromia | Bisidemo | Bisidemo general hospital | 1.5 million |
| 7 | Addis Ababa | Addis Abeba | *Yekatit 12 referal Hospital and **Myungsung Christian Medical Center (MMC) | 3 to 5 million |
| 8 | Drie Dawa | Drie Dawa | Dilechora general hospital | 1.5 million |

*Hospitals with capacity for noma surgery

** Private Hospital

Two of the only NGOs (Project Harar and Facing Africa), involved in Noma management and care in the country has been included in the study. The selection of hospitals and NGOs was done based on convenience, prioritizing hospitals that provide plastic surgery, oral care, and dermatology services in each region. Possible entry points for Noma cases were identified by consulting healthcare workers, including physicians and dental surgeons, and reviewing health facility records.

Comprehensive information regarding Noma surgical management was obtained from the two NGOs' service record books. The record was validated by tracing back the patient records in each hospital where the procedure was performed. All diagnosed Noma cases, including individuals, were included in the study. The collected information from the records encompassed participants' sociodemographic data (name, age, sex, nationality, residence), year of encounter, and any other associated pathologies present at the time of presentation to the health unit.

A database was created including all noma cases found in the hospital register during the visit. In the health facilities a records from 2015 to 2022 was seen. Data collection occurred from September 20 to November 2, 2022. The years were based on availability of records within the health system. Data were collected in person on paper, and then entered into an excel database for analysis.

The national annual Neglected Tropical disease meeting organized in Bahir Dar September 19 to 21, 2022 was used to recruit participants to assess the health workers knowledge and health facility capacity assessment. The conference was hosting about 200 attendants and 40 participants were selected randomly.

We used descriptive analysis to present the result of the quantitative data.

*Qualitative data collection;* Qualitative data was collected through semi-structured interviews with three plastic surgeons involved in Noma management and the surgical campaign, as well as two noma project country directors. A combination of purposive sampling and snowball sampling was employed to select the participants invited for the interviews. Participants were contacted in-person and via email, to ask for their willingness to participate.

All interviews were conducted in-person in English and written-recorded. An interview guide with open-ended questions was developed based on the research objectives, specifically targeting the experience of the experts in noma care, number of cases they have operated, partner organization involved; the role of partner organizations; the type of capacity building which is necessary to enable those institution; the existing strategies policies and plans that could be linked to noma prevention and care; health sector capacities such as health services, information system, financing and human resource capacities and resource needed in case management. Additional probe questions were added to delve deeper into interviewees’ views on and experiences on the main challenges of the expertise and NGO’s working in noma prevention and care in the country and what will be their recommendation to solve those problems.

The qualitative study interview note was converted in edited transcription, where transcriptions were edited to increase clarity and readability. Atlas.ti Version 23.4.0 (29342) was used to support further analysis of the transcripts. A combined inductive and deductive theoretical thematic analysis with open coding was used for data analysis.

To consolidate the knowledge gained by the qualitative and quantitative study a desk review was done to rapidly synthesize existing literature, both peer-reviewed and unpublished from a variety of sources, on case report noma or burden study in Ethiopia, health policies, health professional educations curriculums, guidelines on oral care, and information about the current situation on case reporting system.

The desk review began with searches of academic databases focusing on peer-reviewed literature (Pubmed/Medline, Web of Science and google scholar) and Ethiopian Ministry of Health and NGO’s (who are involved in Noma care) websites and databases. Search terms included “Ethiopia,” “noma,” “health,” “oral health,” “cancrum oris”, “ gangrenous stomatitis”, “gingivitis,” and “Neglected disease ,” “.In addition to these data collection methods, we also elected to contact oral health experts; disease prevention and health promotion directorate; neglected Tropical Disease focal person; NGO’s who are working on noma care, to request additional resources for inclusion in the desk review that may not have been available through one of the aforementioned databases. Literatures and documents written in English and Amharic has been included in the review. The finding ware documented in thematic area burden of disease, availability of policy framework in Noma prevention and care and health system reediness.

**Main findings**

**Stakeholder analysis**

The Ethiopian MOH and the Regional health bureaus are the main players of the health system in designing a policy, developing strategic plans, budgeting and cascading  implementation of any health program including Noma.

In the  stakeholder analysis the assessment found two hospitals, two NGOs and one rehabilitation center actively involving in Noma case finding, clinical care and rehabilitation.

*Notable Stakeholders:*

- Project Harar**:** is a charity registered in England and Wales and in Ethiopia. At the binging (2003) the organization was helping noma patients in getting surgery in UK but letter on the project expanded to any facial disfiguring diseases which needs surgical intervention. Since 2004 the organization has developed a unique outreach model to find patients from even the most remote rural areas, and to transport them to Addis Ababa for the surgery. Project Harare is working specifically with Yekatit 12 Hospital in Addis Ababa organize the surgical campaign.
- Facing Africa: A UK Registered charity with office in Ethiopia started working in 2007 in noma care and other facial deformities which need surgical intervention. Since 2007 Facing Africa fund and organises two bi annual surgical trips to Ethiopia independently. The main activity of Facing Africa is in fund raising to support teams of professionals each year from Europe to Ethiopia to cover air fares. The organization also uses local experts (plastic surgeons) and collaborate with two Hospitals in Addis Ababa (Yekatit 12 Hospital and Myungsung Christian Medical center) to perform the operation

*Key Hospitals:*

- Yekatit-12 Hospital, Addis Ababa: is a public teaching hospital in Addis Ababa and is a key partner in providing noma and cleft surgery for Project Harar’s patients and Facing Africa. Each year the hospital hosts one to two complex surgical missions. UK, international and Ethiopian medical staff work together at Yekatit-12 to provide surgery to patients with complex facial conditions.
- Myungsung Christian Medical center (Korea Hospital): is a missionary general hospital in Addis Ababa. The hospital offers plastic surgery and maxillofacial surgery. The hospital works with Facing Africa in providing surgical service for noma patients
- Tibebe Ghion Specialized Hospital: a public teaching Hospital of Bahir Dar University, in Amhara region, Bahir Dar. The Hospital has a head and neck surgeon who is performing noma surgery integrated in the routine care (Figure 2).

**Policy**

The Federal Democratic Republic of Ethiopia put in place the National Health Policy and Strategy in 1993. The development of Health Sector Development Plan (HSDP) of the National Growth and Transformation Plan (GTP) and Health Sector Transformation Program (HSTP) implemented the policy throughout the country. In the last two decades different initiatives including hospital reforms has been implemented to improve services as well as pro-poor interventions to reorient health services towards health promotion, disease prevention, curative services throughout the country(19).

Because of these initiatives currently the country is well placed in achieving primary health care coverage. Ethiopia has achieved substantial progress in improving many key health indicators during the past two decades.

The major health problems of the country remain largely preventable communicable diseases and nutritional disorders. Malnutrition and WaSH, are considered as the top two risks that drove the most deaths and disabilities in the country(18).

There is no standalone national policy on noma or NTDs in Ethiopia. However, the health policy of the country emphasizes the prevention and control of diseases of poverty.

***Health work force*:** In the country health professional training initiative dental care program was expanded from one public centre in Addis Ababa to multiple both public and private schools both in Addis and in the regions. The programs delivers training for Dental professionals, Dental surgeons and maxillofacial surgery. But there is no program for training of dental hygienist/therapist or dental practitioner/nurse. The last national human resource strategic plan (2016-2025) in its health facility standard projected to assign at list one dental hygienist at health canter level to prevent and treat oral diseases as well as to promote oral health need. Despite all the last report (2016) the number of dental professional including dentists and dental hygienists working in the country health system was only 270. The strategic plan has also projected to assign minimally two dental professionals (BSc) to primary hospital, but the assessment could not find record or other evidences on the implementation of the plan (19). The Inservice curriculum for Dental doctors includes noma and its prevention and management. Inservice training Dental care training is also included in doctor of medicine and Nursing curriculum with one credit hour. Currently there is no pre-service training or continuous professional development for oral/dental care in the country.

***Health information system:*** The Ministry of Health has also put in place HMIS to gather data from all health facilities in the country. The system is in its scale-up phase and need to be expanded to address diseases of the poor. Currently the system does not include the indicators to acute cases and sequel of noma.

***Literature reviews***: The first report of noma cases in Ethiopia who has received reconstructive surgery from medical mission (project Harar) was from 2007 to 2009 which has reported 43 cases. The report emphasized the similarity of the gross disease encountered to that faced by surgeons in the nineteenth century. The article has also advocates for teams of healthcare workers to move to areas of need rather than sending patients abroad for the surgery (15). The second report by another medical mission (Facing Africa) was a retrospective review of 81 cases who has undergone reconstructive surgery from 2015 to 2019. The report uncovered critical information’s such as age of onset (5 year and 8 months), median time of presentation (18 years from onset to accessing treatment), geographic distribution of patients, and significantly decreased patient quality of life (14).

A retrospective study to assess the clinical presentation of patients in 161 noma patients registered in three centres in Addis Ababa. The facial defects ranged from minor to severe tissue damage with 19.3% grade 1 (0-25% tissue damage), 42.3% reported as grade 2 (with 25 to 50% tissue damage), 30.7% grade 3 (50 to 75% tissue damage) and 8.1% grade 4 (75 to 100% tissue damage). The commonest anatomic region affected by the disease are cheek, upper lip, lower lip, nose, hard palate, maxilla, oral commissure, zygoma, infra-orbital region, mandible, and chin (21).

**Health care workers knowledge and health sector capacity assessment**

Forty federal and regional health system leaders, experts, and NGOs who are working on NTDs responded for the rapid assessment on knowledge, practice and Information about noma. Among the participants, 27 were public health professionals and specialists, nine medical doctors, two laboratory technologists, two health care administrators. The mean experience in the health system was 18 years (ranging 2 to 45 years). The participants’ roles varies from NTD experts and researchers to directors working in public sector and NGOs.

Fifteen (37.5%) participants reported having heard about noma, but only 6 (40%) of those with prior information have seen a case. Eighty-five percent of the participant stated that there is no oral health program in the country’s health system. All participant agreed on the limitation of on the availability of human resource for oral health and poor capacity of the health institution for integrating of oral health program in to the primary health care unit.

Sixty percent of respondents said noma can be integrated into NTD programs, while 40% preferred integration into Non Communicable Disease, nutrition and medical services.

For the key informant interviews, three plastic surgeon with 10 to 15 years of experience working on noma were selected. Two NGO’s country directors, who have been working on noma for 5 to 7 years, were also interviewed. A plastic surgeon performing constructive surgery stated, “In the last 10 years, I have seen about 300 cases, but since 2020, we are seeing fewer noma cases. However considering the country situation with war and drought, it is expected that noma cases will increase in the coming years. We have not operated on noma patients for the past two years due to COVID 19 pandemic movement restriction.”.

Regarding noma prevention and the surgical intervention, another plastic surgeon responded, “Noma prevention activities do not exist until now, but it is very important to initiate and develop a prevention system, especially at this time, because the ongoing war and drought will likely increase the number of noma patients. Prevention should focus on both primary and secondary levels to prevent the occurrence of the disease and minimize complications and suffering. Activities should include supporting existing noma management centers, establishing new centers at different levels, creating awareness, training professionals, procuring instruments, and securing financial resources to support and upgrade available centers.

“The surgery for noma is demanding because the destruction caused by the disease is usually extensive and involves vital body parts. The patients' nutritional status is typically very poor, and the surgery is comprehensive, staged, demanding, and requires the expertise of various professionals. To achieve good surgical outcomes, patients need preoperative and postoperative rehabilitation, including nutritional, psychological, physical, and occupational therapy. Unfortunately, these services are not available in the routine health system.”

A head and neck surgeon from Bahir Dar with experience in performing complex facial tumor and noma surgeries stated, “We have performed some stage 5 noma surgeries in the routine system by Ethiopian surgeons with successful outcomes. We are training surgeons at St. Paul Millennium Hospital and Medical College to perform these surgeries” (Figure 2).

Figure 2. A 22 year old male patient (before surgory and two weeks after surgory), surgory performed in the routine program in Tibebe Ghion Specialized Hospital, Bahir Dar, Ethiopia (Photo courtsy of Melesse Gebeyehu, 2022)

A key informant from an NGO working on recruiting patients and supporting public hospitals for surgical management, when asked about the type of capacity building necessary, stated, “Training for plastic surgeons (residency) and equipment are the main areas of support. Due to the complicated nature of the surgery, postoperative services are very important.” Regarding the main challenges faced by NGOs working on noma patient care, they noted, “Equipment, pre- and post-operative care and treatment accommodation, medication, human resources, timely approval of medical team credentials by the Ethiopian Food and Drug Authority, ongoing training for the local team on case management, easy and affordable/subsidized diagnostic centers, follow-up of cases after discharge, and the availability of pre- and post-operative accommodation centers for patients and parents. Identifying cases is also a challenge that I encountered during my time on the project.”

**A retrospective assessment of noma cases**

In total the assessment have extracted 69 completed record reported from January 2015 to December 2020 (Figure 3). 38 (55.1%) of cases are female and 44.9% of cases are male. The mean age of patient is 28 years (ranging 4 to 70 years). The recorded patient address indicated that 31 patients are from Amhara region, 9 from Somalie, 8 from Oromia, 5 from Afar, 5 from SNNPR, 4 from Gambella and 3 from Addis Ababa. Most of the report (67 patients) were extracted from the NGO data base who are working on noma care. Two patients the surgery was performed by in Bahir Dar by Ethiopian Head and neck surgeon in the routine care program without the assistance of the NGOs .

Figure 3. Trend of noma cases who have undergone reconstructive surgery in Ethiopia from 2015 to 2020.

Two of the patients with address in Gambela region are from Sudanese refugee camp. There is no record for patient clinical presentation, duration of illness, severity and patient outcome.

A study from Ethiopia on the risk factor has also concluded poverty-related diseases such as malaria, helminths infection, measle, diarrheal diseases, and unfavourable living conditions were identified to be the risk factor for noma. As such the disease is truly preventable. Prevention of the disease can be achieved through promoting overall awareness of the disease, poverty reduction, improved nutrition, and promotion of exclusive breastfeeding in the first 3-6 months of life. Furthermore, optimum prenatal care, timely immunizations against common childhood diseases, initiating vaccination, and improving the social living conditions are the other preventive mechanisms. Moreover, long-lasting economic development should be considered to effectively and sustainably prevent the disease.

The mean self-reported age of onset was 5 years and 8 months, with a median time of 18 years from onset to accessing treatment. Before intervention, 65% covered their face in public, 59% reported difficulty eating, 81% were unhappy with their appearance, and 71% experienced bullying.

**DISCUSSION**

The assessment showed that the country is endemic for noma and cases are reported from all corner of the country with regional variation in case distribution with the highest case coming from Amhara region (45%) followed by Somali region (13%). The strong focality of noma cases in Amhara and Somali region also goes also with the highest prevalence of childhood malnutrition on those regions (21). Not finding any record of acute cases, a calls for urgent development of epidemiological surveillance programs and improving the HMIS/DHIS indicators to document noma. More accurate data are needed and for this it is important to develop a routine program for identifying and recording cases of noma at the primary health facility level.

The rapid assessment identified that a record of noma patient in Ethiopia in the last two decades. The trend of cases being cared has decreased from 2015 to 2020 but considering the surgery being done in campaign with a support from missionaries, the conclusion may be misleading. Previous studies in the country showed that the mean age of onset to be 5.6 and median age at presentation 25 which is in agreement with our finding . The median time from onset of the disease to access of treatment of noma patient in the country is 18 years (14). The delay on access for treatment is the reflection poor access to specialist health care in the region. The rapid assessment have also identified only three hospitals (two in Addis Ababa and one in Bahir Dar) are managing noma patients and the hospitals in Addis Ababa provide service only when there is medical mission campaign. Two studies on the clinical presentation of noma patients in the country showed that most of the patients suffered from extensive noma-induced facial disfigurements, and functional deficit with significant social discrimination, and negative psycho-social effects (14, 20).

A study in the country on the risk factor of noma has also recommended prevention of the disease can be achieved through promoting overall awareness of the disease, poverty reduction, improved nutrition, and promotion of exclusive breastfeeding in the first 3-6 months of life. Furthermore, optimum prenatal care, timely immunizations against common childhood diseases, initiating vaccination, and improving the social living conditions are the other preventive mechanisms. Moreover, long-lasting economic development should be considered to effectively and sustainably prevent the disease (20).

In the last report of Human resource for health (2016) in Ethiopia the number of dental professional working in the country health system was only 270, that is one dental professionals to more than 300000 population. The HR strategic plan in its projection has estimated 6576 and 10017 dental professionals in 2020 and 2025 subsequently but the implementation of this plan is not documented (19). In Ethiopia in addition to the limited distribution of oral health personnel, lack of equipped and functional facilities within the primary health care system, the majority of the population have only limited or no access to professional oral health care services. This results in a high proportion of untreated oral diseases and significant needs and demands for essential oral health care services, thus posing challenges to primary health care systems in the country.

The strength of the study is that, this is the first study to document regional distribution of noma cases. The study also analysed the health system strength and gap which will inform policy makers for noma prevention and control program in the country.

The main limitations of the assessment are: Difficulty accessing information from hospitals and NGOs working on noma; lack of source documents at a missionary hospital performing noma surgeries and no records at a rehabilitation center for noma patients; Existing records contain only limited information about individual noma patients; No corroborating records of noma cases reported by physicians; No records of acute noma cases found in hospital HMIS; The assessment did not extensively evaluate existing laws, regulations, policies, and strategies addressing noma.

**Conclusion and Recommendations**

The assessment showed that noma cases are reported from all corner of the country, with regional variation in case distribution. The lack of record for acute cases calls for urgent development of epidemiological surveillance programs and improving the HMIS indicators to document noma. More accurate data are needed, which necessitate developing a routine program for identifying and recording cases of noma at the primary health care level.

This rapid surveillance has identified a striking scarcity of research and surveillance programmes considering noma, with no data on acute cases. Addressing noma effectively will require its inclusion in the national list of neglected tropical diseases and the implementation of integrated control programs aimed at its elimination.

Intensify efforts in terms of public awareness and education of community workers and health professionals at existing health centers on identification of noma (the stages of clinical presentation) and subsequent referral of patients to referral hospitals.

Considering the country situation in the last two years (war, pandemic, drought and health system disruption) and the report that the interruption of medical mission, active surveillance is needed to identify both acute cases and those with scaring disfigurement who needs reconstructive surgery.

We recommend that more research be implemented that includes data collection at the district health facility level to determine the true burden of noma and that prospective studies be implemented to better understand the sequence of events that contribute to the development of noma. With these sets of indicators it will be possible to better formulate and target prevention programs.

With the existing limited number of dental professionals in the country, it will be unrealistic to rely on workforce models to deliver professional treatment at primary healthcare level. One opportunity is to explore the existing health extension program which is the main workforce for the primary health system. Expand oral health services by integrating them into school health programs and the existing NCD or NTD prevention and control programs in the primary health system are also another opportunities to explor.

By following these recommendations, it will be possible to develop effective policies and programs that address noma, improve oral health services, and ultimately contribute to the elimination of this neglected disease.

**Acknowledgment:**

Ministry of Health of Ethiopia for the guidance and administrative support

Project Harar and Facing Africa for sharing data of the Noma cases supported by their mission

Dr Mekonnen Eshete and Dr Melesse Gebeyhu , for their expert input in to the inception of the assessment, providing me information and contact of the stakeholders and their guidance in understanding the Noma surgical care delivery in the country.

To Alemayehu Bekele for his hard work on field based data collection from the selected institution.

And special thanks to WHO country office (Dr Zeyede Kebede, Dr Nigus Manaye, Dr Worku Bekele) and WHO Regional Office for Africa (Dr Yuka Makino) for their guidance, expert advice from the inception to finalization of the field work.

And to all those who directly or indirectly gave their contribution to this work.

**REFERENCE**

1.	World Health Organization Regional Office for Africa. Information brochure for early detection and management of Noma. 2017. Available from <https://apps.who.int/iris/handle/10665/254579>; 2017. Available: <https://apps.who.int/iris/handle/10665/25457979%0AAdditional>.

2.	Ashok N, Tarakji B, Darwish S, Rodrigues JC, Altamimi MA. A Review on Noma: A Recent Update. Global journal of health science. 2015;8(4):53-9.

3.	Bourgeois DM, Leclercq MH. The World Health Organization initiative on Noma. Oral diseases. 1999;5(2):172-4.

4.	Baratti-Mayer D, Pittet B, Montandon D, Bolivar I, Bornand JE, Hugonnet S, et al. Noma: an "infectious" disease of unknown aetiology. The Lancet Infectious diseases. 2003;3(7):419-31.

5.	Baratti-Mayer D, Gayet-Ageron A, Hugonnet S, François P, Pittet-Cuenod B, Huyghe A, et al. Risk factors for Noma disease: a 6-year, prospective, matched case-control study in Niger. The Lancet Global health. 2013;1(2):e87-e96.

6.	Tempest MN. Cancrum oris. The British journal of surgery. 1966;53(11):949-69.

7.	World Health Organisation (WHO) and Hilfsaktion Noma. Promoting Oral Health in Africa: Prevention and control of oral diseases and Noma as part of essential noncommunicable disease interventions. 2016. Available: <http://www.aho.afro.who.int/en/news/5236/official-launch-who-manual->“promotingoral-health-africa”.

8.	Srour ML, Watt B, Phengdy B, Khansoulivong K, Harris J, Bennett C, et al. Noma in Laos: stigma of severe poverty in rural Asia. The American journal of tropical medicine and hygiene. 2008;78(4):539-42.

9.	Farley E, Mehta U, Srour ML, Lenglet A. Noma (cancrum oris): A scoping literature review of a neglected disease (1843 to 2021). PLoS neglected tropical diseases. 2021;15(12):e0009844.

10.	Leila Srour M, Marck KW, Baratti-Mayer D. Noma: neglected, forgotten and a human rights issue. International health. 2015;7(3):149-50.

11.	World Health Organization, OMS. World Health Report Life in the 21st century A vision for all Report of the Director-General. 51st World Heal Assem. 1998; 1–226. Available: <https://www.who.int/whr/1998/> en/whr98_en.pdf?ua=1.

12.	Bourgeois DM, Diallo B, Frieh C, Leclercq MH. Epidemiology of the incidence of oro-facial Noma: a study of cases in Dakar, Senegal, 1981-1993. The American journal of tropical medicine and hygiene. 1999;61(6):909-13.

13.	Farley E, Oyemakinde MJ, Schuurmans J, Ariti C, Saleh F, Uzoigwe G, et al. The prevalence of Noma in northwest Nigeria. BMJ global health. 2020;5(4):e002141.

14.	Rickart AJ, Rodgers W, Mizen K, Merrick G, Wilson P, Nishikawa H, et al. Facing Africa: Describing Noma in Ethiopia. The American journal of tropical medicine and hygiene. 2020;103(2):613-8.

15.	McGurk M, Marck R. Treatment of Noma: medical missions in Ethiopia. British dental journal. 2010;208(4):179-82.

16.	Ethiopia Overview. <https://www.worldbank.org/en/country/ethiopia/overview>. Accessed November 07.

17.	The Federal Democratic Republic of Ethiopia MoHSEHA, 2016/17. Addis Ababa, Ethiopia. 2019.

18.	FMOH, User Guide for Investment in Health Sector. Addis Ababa: MOH Federal Democratic Republic of Ethiopia Ministry of Health J, 2019..

19.	FMOH. Ethiopian Health Sector Transformation Plan. 2015/16-2019/20. Federal Femocratic Republic of Ethiopia Ministry of Health.2015;20(May)

20.	Gebretsadik HG, de Kiev LC. A retrospective clinical, multi-center cross-sectional study to assess the severity and sequela of Noma/Cancrum oris in Ethiopia. PLoS neglected tropical diseases. 2022;16(9):e0010372.
