# Supplementary material for: Rapid assessment of noma: Reporting on forgotten and neglected disease in Ethiopia
Source: PLoS Negl Trop Dis. 2024 Dec 13;18(12):e0012696. doi: 10.1371/journal.pntd.0012696 (PMC11676800; doi:10.1371/journal.pntd.0012696)
Supplement: Attachment — Submitted filename: Response to reviewer 10112024.docx [file pntd.0012696.s004.docx]

Response to reviewer 10112024

1. The authors contribution is added in the first page (title page)

2. The result is added and subsection are restructured based on the guideline

3. Legend for supporting information is added after the reference

4. Any patient identifiers data is removed and the revised databases is uploaded

5. For one of the images(patient) a signed copy right permission is obtained (uploaded)

Figure 1 map source attribution was added in the legend

Figure 2 Image additional black line was added to cover the eye and nose. The patient is Ethiopian and he signed the consent form in the language he can understand but I have added also the English translation based on our previous editorial communication
